# Genome Sequence of Avirulent *Riemerella anatipestifer* Strain RA-SG

**DOI:** 10.1128/genomeA.00218-12

**Published:** 2013-03-07

**Authors:** Jianfeng Yuan, Linlin Li, Minhua Sun, Jiawen Dong, Qilin Hu

**Affiliations:** Institute of Veterinary Medicine, Guangdong Academy of Agricultural Sciences, Guangzhou, China, and Guangdong Open Laboratory of Veterinary Public Health, Guangzhou, China

## Abstract

*Riemerella anatipestifer* is a pathogenic bacterium that has spread all over the world and is associated with epizootic infections in waterfowl and other avian species. *R. anatipestifer* RA-SG is an avirulent strain, isolated from an infected duck in Guangdong province, China. The genome sequence of this species is presented herein.

## GENOME ANNOUNCEMENT

*Riemerella anatipestifer*, a Gram-negative, gliding-motile, and non-spore-forming rod-shaped bacterium, is the etiological agent of a contagious septicemic disease in waterfowl and other avian species (1, 2); according to 16S rRNA gene analysis, it belongs to the family *Flavobacteriaceae* (3, 4). Up to now, at least 21 serotypes have been identified by tube agglutination and agar gel precipitin tests, and no cross-immunoprotection has been reported (5, 6). Based on public papers, three genome sequences of *R. anatipestifer* have been reported until now, those of strains ATCC 11845, RA-GD, and RA-YM (7–10). All of them are highly virulent to the susceptible hosts. Here, we sequenced the genome of *R. anatipestifer* RA-SG, an avirulent strain. We believe that it will be very helpful in understanding further the pathogenesis of *R. anatipestifer*.

The genome sequence was determined using an Illumina genome analyzer (0.5-kb and 2-kb paired-end libraries), with sequencing carried out by the Beijing Genomics Institute (BGI) in Shenzhen, China. The sequence reads were assembled into a total of 75 contigs (N_50_ length, 224,073 bp) distributed over 31 scaffolds (N_50_ length, 436,370 bp) using Short Oligonucleotide Alignment Program (SOAP)denovo. Glimmer 3.0 was used to predict the protein-coding sequences (CDSs) (11). The tRNAs, rRNAs, and small RNAs (sRNAs) were identified using tRNAscan, rRNAmmer, and Rfam, respectively (12–14). Genome annotation was generated by searching against the NCBI nonredundant (NR), Swiss-Prot, TrEMBL, Clusters of Orthologous Groups (COGs), and Kyoto Encyclopedia of Genes and Genomes (KEGG) databases (15, 16).

The genome sequence of avirulent *R. anatipestifer* RA-SG comprises 2,172,634 bp, with an average G+C content of 34.94% and a coding percentage of 90.37. The genome contains 2,072 putative open reading frames with an average length of 948 bp; it also contains 36 tRNA genes, 4 rRNA genes, and 9 sRNAs, constituting 90.37% of the genome. Of the CDSs, 37.55% could be assigned to COG families, and 959 genes were designated to 129 KEGG pathway mappings using KEGG Automatic Annotation Server (KAAS) (17). The repeat sequence length of 16,601 bp was determined by the RepeatMasker, RepeatProteinMasker, and Tandem Repeats Finder software programs. In addition, there are two credible clustered regularly interspaced short palindromic repeats (CRISPRs) and two possible CRISPRs, but there is no prophage sequence in the genome. Except for the general secretory (Sec) and twin-arginine translocation (Tat) pathways, the classic secretion systems in the Gram-negative bacteria were not identified, which has been reported in other virulent *R. anatipestifer* strains. Moreover, three genomic islands were identified using SIGI-HMM; these were 4,772 bp, 3,537 bp, and 8,289 bp.

Overall, the genome sequence of avirulent *R. anatipestifer* RA-SG will provide the basis for a better understanding of the molecular pathogenesis of *R. anatipestifer* using comparative genomics.

### Nucleotide sequence accession numbers.

This Whole Genome Shotgun project has been deposited at DDBJ/EMBL/GenBank under the accession no. ANGF00000000. The version described in this paper is the first version, ANGF01000000.
